# IMPLICATIONS OF RELATIONSHIPS WITH FAMILY, FRIENDS, AND NEIGHBORS FOR WOMEN'S WELL-BEING IN LATE LIFE

**DOI:** 10.1093/geroni/igz038.778

**Published:** 2019-11-08

**Authors:** Heather Fuller, Heather Fuller, Masahiro Toyama

**Affiliations:** North Dakota State University, Fargo, North Dakota, United States

## Abstract

Social support is well documented as promoting women’s well-being across the lifespan, yet implications vary depending on the source and type of support. The present study examined whether relationships with family, friends, and neighbors (both satisfaction with and number in social network) affected well-being over two years. Midwestern women (N=188, mean age = 80) were sampled from two waves of the Social Integration and Aging Study (2013, 2015). Hierarchical regression models indicated that satisfaction with friends predicted better life satisfaction, but satisfaction with family and neighbors did not predict well-being. In contrast, number of neighbors in social network predicted lower life satisfaction and greater stress, while number of family and friends were not associated with well-being. Moreover, differential effects emerged between older and younger women. Findings highlight unique facets of older women’s social relationships and suggest that future research and interventions addressing age and the source of support are warranted.

